# Smoking, once again

**DOI:** 10.4103/1817-1737.74278

**Published:** 2011

**Authors:** Abdullah Al-Mobeireek

**Affiliations:** *King Faisal Specialist and Research Centre, Al Shoura Council Member, Health Committee, Riyadh, Saudi Arabia. E-mail: mobeireek@yahoo.com*

Sir,

Commentary on Tobacco consumption: Is it still a dilemma?

I thank Dr. Al Moamary for designating an editorial and ringing the bell on the smoking epidemic in the Kingdom of Saudi Arabia (KSA).[1] The smoking statistics that he has quoted are indeed worrying. Strikingly, although KSA has a population over 20 million, it is the fourth in the world in terms of tobacco consumption and sales. Health institutions are already receiving a significant number of smoking victims and are expected to be overwhelmed when the epidemic manifests itself as the current smoking population advances in age.

More is needed to control the smoking epidemic, in particular, enforcing the smoking ban, raising the taxes (which is one of the lowest in the world), and pursuing the tobacco companies legally.

The objectives of smoking ban in public places is to protect non-smokers (innocent bystanders) from risks of second hand smoking (SHS), and to make smoking more difficult for smokers which may help them to quit or at least reduce smoking. As far as SHS is concerned, more worrying stastics have come recently from WHO showing a yearly death toll over 600, 000 worldwide, 165, 000 of which are amongst children.[2]

I would like here to complement Dr. Al Moamary’s editorial, by clarifying the situation of the smoking ban in KSA. Although the anti-smoking law in KSA was passed nearly 10 years ago by Al Shoura Council,[3] unfortunately it has not been yet activated. Only recently (November 2010), the smoking ban was enforced in airports, which is a positive step forward. Some places, such as King Saud University and few shopping malls have voluntarily established their own anti-smoking policies, a step they should be thanked for. However, the force of law is yet to reach thousands of public places such as restaurants, coffee shops, parks, and work places where smoking goes on unnoticed.

Physicians, especially in thoracic medicine, are the most able people to appreciate the considerable suffering that victims of smoking endure, as well as the health and economic burden on the country. Therefore, they should take an active role in the tobacco control. This year (2010) has been declared as the year of the lung by a number of international respiratory societies.[4] Through their professional societies, in particular, the Saudi Thoracic Society (STS) and Saudi Heart Association (SHA), physicians in KSA should lobby to enforce the anti-smoking law in public places and support all other measures to control this epidemic. Those involved in academic institutions need to engage in more research on all aspects pertaining to smoking, whether it is epidemiological surveys, psychology of the addiction, or newer therapies, including vaccines. With the preponderance of research chairs at universities nowadays, I dream of a research chair designated to tackle smoking.
